# Susceptibility of adult female *Aedes aegypti *(Diptera: Culicidae) to the entomopathogenic fungus *Metarhizium anisopliae *is modified following blood feeding

**DOI:** 10.1186/1756-3305-4-91

**Published:** 2011-05-26

**Authors:** Adriano R Paula, Aline T Carolino, Carlos P Silva, Richard I Samuels

**Affiliations:** 1 Department of Entomology and Plant Pathology, Universidade Estadual do Norte Fluminense Darcy Ribeiro, Campos dos Goytacazes, RJ 28013-602, Brazil; 2 Departamento de Bioquímica, Universidade Federal de Santa Catarina, Florianópolis, 88040-900, Brazil

**Keywords:** *entomopathogenicfungus*, virulence, blood feeding, insect, vector, dengue, *Metarhiziumanisopliae*, *Aedes aegypti*

## Abstract

**Background:**

The mosquito *Aedes aegypti*, vector of dengue fever, is a target for control by entomopathogenic fungi. Recent studies by our group have shown the susceptibility of adult *A. aegypti *to fungal infection by *Metarhizium anisopliae*. This fungus is currently being tested under field conditions. However, it is unknown whether blood-fed *A. aegypti *females are equally susceptible to infection by entomopathogenic fungi as sucrose fed females. Insect populations will be composed of females in a range of nutritional states. The fungus should be equally efficient at reducing survival of insects that rest on fungus impregnated surfaces following a blood meal as those coming into contact with fungi before host feeding. This could be an important factor when considering the behavior of *A. aegypti *females that can blood feed on multiple hosts over a short time period.

**Methods:**

Female *A. aegypti *of the Rockefeller strain and a wild strain were infected with two isolates of the entomopathogenic fungus *M. anisopliae *(LPP 133 and ESALQ 818) using an indirect contact bioassay at different times following blood feeding. Survival rates were monitored on a daily basis and one-way analysis of variance combined with Duncan's *post-hoc *test or Log-rank survival curve analysis were used for statistical comparisons of susceptibility to infection.

**Results:**

Blood feeding rapidly reduced susceptibility to infection, determined by the difference in survival rates and survival curves, when females were exposed to either of the two *M. anisopliae *isolates. Following a time lag which probably coincided with digestion of the blood meal (96-120 h post-feeding), host susceptibility to infection returned to pre-blood fed (sucrose fed) levels.

**Conclusions:**

Reduced susceptibility of *A. aegypti *to fungi following a blood meal is of concern. Furthermore, engorged females seeking out intra-domicile resting places post-blood feeding, would be predicted to rest for prolonged periods on fungus impregnated black cloths, thus optimizing infection rates. It should be remembered that lowered susceptibility was only a temporary phenomenon and this may not necessarily occur when mosquitoes are infected with other fungal isolates. These results may have implications for field testing of entomopathogenic fungi by our group and further studies should be carried out to better understand the insect-fungus interaction.

## Background

Dengue fever (DF) and the potentially lethal version of the disease, dengue haemorrhagic fever, which has now been detected in almost all countries where DF is prevalent, has become a major cause of hospitalization and death among children and the old. Approximately 2500 million people, two fifths of the world's population, are now at risk from DF. The WHO currently estimates there may be 50 million cases of DF infection worldwide every year [1]. Vaccine development is still at an early stage and therefore the only method available for reducing incidence of the disease is the control of its mosquito vector.

Current strategies based on the elimination of breeding sites and applications of chemical insecticides for larval and adult mosquito control have resulted in development of resistance without eliminating the constant risk of dengue epidemics [2]. Thus new approaches are urgently needed.

A wealth of studies has shown the potential of entomopathogenic fungi for the control of the malaria mosquitoes *Anopheles gambiae *and *An. stephensi *[3-9]. However, the dengue vector *Aedes aegypti *has not received the same attention, although it is also susceptible to infection by entomopathogenic fungi. Scholte et al. [10] showed that an isolate of *Metarhizium anisoplaie *was pathogenic to adult *A. aegypti *and *Aedes albopictus *, vectors of dengue fever and yellow fever. Paula et al. [11] screened a range of *Beauveria bassiana *and *M. anisopliae *isolates for virulence against *A. aegypti *and more recently Paula et al. [12] used a combination of *M. anisoplaie *with low concentrations of an insecticide, Imidacloprid, to reduce survival rates following short exposure periods to the two agents. It has also been shown that *A. aegypti* males contaminated with *B. bassiana* transmitted conidia to females following copulation, resulting in infection of the females [13].

Importantly, previous studies have shown that during the infection process, entomopathogenic fungi can modify host physiology and behavior, for example blood feeding propensity by female insects was reduced following fungal infection of *An. stephensi *[4] and *An. gambiae *[14]. Fecundity was also negatively affected during fungal infections of *An. gambiae *[14] and *A. aegypti *[13]. This means that a reduction in "vectorial capacity" can occur even before the mosquitoes die, an extra benefit when using this type of approach.

Conversely, the physiological state of the target insect may alter its susceptibility to microbial infection. Studies have shown that anautogeny, the condition where gravid females need to feed before oviposition, in this case on blood, infers greater fitness to *A. aegypti *when compared to those fed on sucrose [15], although a recent study showed that blood feeding alone reduced survival rates when compared to sucrose or sucrose + blood fed *An. gambiae *[16].

In the study of reduced malarial transmission by fungus infected *An. stephensi *, Blanford et al. [4] showed that female mosquitoes exposed to fungal conidia immediately after a blood meal had lower survival rates than females exposed to conidia three days after a blood meal. This correlation of reduced susceptibility with time after feeding was shown for up to 12 days following the blood meal. However, the authors did not discuss the importance of this data. Interestingly, a study on the effects of age and blood feeding on fungal infection of *An. gambiae *, showed higher survival rates of blood fed females when compared to glucose fed counterparts [17].

Here we document the changes in susceptibility of *A. aegypti *to fungal infection by *M. anisopliae *following blood feeding and discuss the possible causes and implications of this phenomenon for the biological control of this important vector species.

## Results

**Figure 1:**
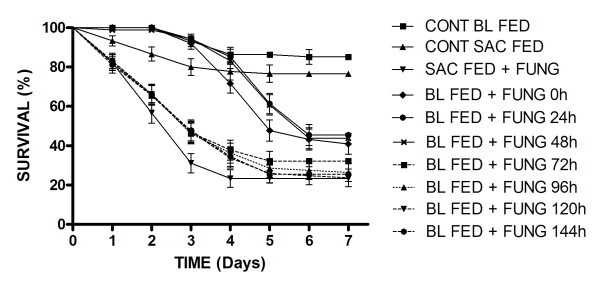
**Survival curves of blood or sucrose fed female *Aedes aegypti *(Rockefeller strain) exposed to *M. anisopliae *LPP 133 at different times post-feeding**. The control survival curve (Control BL FED) is the mean of the controls for each blood fed group (7 groups) for each time period post-feeding. Results are the means of three experiments for each treatment with 20 insects used per experiment. Data points without standard error bars had errors equal to that of the previous data point.

All treatment groups exposed to fungi had reduced survival rates when compared to control groups not exposed to fungal conidia. However, a significant alteration in susceptibility was seen immediately following blood feeding for the Rockefeller and wild strain insects exposed to both isolates tested here. When comparing mean percentage survival of sucrose and blood fed females exposed to fungi, significant differences were seen from time zero up to 72 h post-feeding. In the case of Rockefeller strain exposed to LPP 133, a one-way analysis of variance showed a significant difference between blood fed and sucrose fed insects (F_(9,29) _= 141.41; *p *<0.01; Table 1). When using pair-wise comparisons of sucrose fed and blood fed females at time zero, the survival curves (Figure 1) were significantly different (χ^2 ^35.66; df = 1; *P *< 0.0001). This was also the case for pair-wise comparisons (24, 48 h) until 72 h post-blood feeding, when the survival curves were not significantly different (χ^2 ^2.87; df = 1; *P *= 0.0903).

**Table 1:** Survival of female *A. aegypti *(Rockefeller strain) fed on sucrose or blood and subsequently exposed to fungal isolates at different times post feeding

Treatments	LPP 133		ESALQ 818	
	**% Mean Survival ± SD **	**S_50 _**	**% Mean Survival ± SD **	**S_50 _**
CONT SUC	76.6 ± 2.88 a	ND	73.3 ± 2.50 a	NA
CONT BL	85.5 ± 2.55 b	ND	77.7 ± 2.06 a	NA
SUC FED + FUNGUS	23.3 ± 10.66 e	3	25 ± 8.96 d	3
BL FED 0 h + FUNGUS	42.2 ± 8.63 c	5	43.3 ± 7.13 b	6
BL FED 24 h + FUNGUS	46.6 ± 8.23 c	6	42.2 ± 6.50 b	6
BL FED 48 h + FUNGUS	44.4 ± 8.00 c	6	45 ± 7.15 b	6
BL FED 72 h + FUNGUS	31.1 ± 7.58 d	3	36.6 ± 6.30 bc	4
BL FED 96 h + FUNGUS	27.7 ± 7.43 de	3	31.1 ± 6.89 cd	3
BL FED 120 h + FUNGUS	26.6 ± 7.16 de	3	27.7 ± 8.51 d	3
BL FED 144 h + FUNGUS	25.5 ± 6.16 e	3	22.1 ± 7.98 d	3

Note: following the initial blood feed, mosquitoes were offered sucrose on a daily basis.

Results followed by the same letter indicate no significant differences when using Duncan's *post-hoc *test (5% probability). Values for S_50 _were calculated using Kaplan-Meier survival analysis. CONT: control; SUC: sucrose; BL: blood; NA: not applicable.

**Figure 2:**
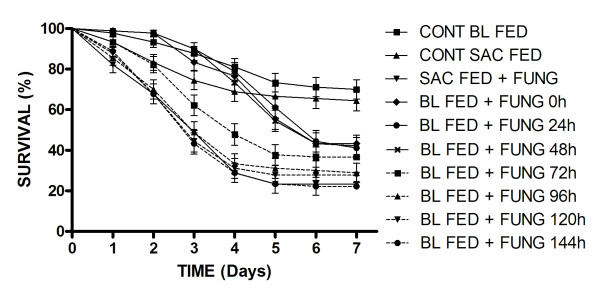
**Survival curves of blood or sucrose fed female *Aedes aegypti *(Rockefeller strain) exposed to *M. anisopliae *ESALQ 818 at different times post-feeding**. The control survival curve (Control BL FED) is the mean of the controls for each blood fed group (7 groups) for each time period post-feeding. Results are the means of three experiments for each treatment with 20 insects used per experiment. Data points without standard error bars had errors equal to that of the previous data point.

A similar pattern was also seen for insects exposed to isolate ESALQ 818 (F_(9,29) _= 54.17; *p *< 0.01; Table 1). When using pair-wise comparisons of sucrose fed and blood fed females at time zero the survival curves (Figure 2) were significantly different (χ^2 ^30.05; df = 1; *P *< 0.0001). This was also the case for pair-wise comparisons at 24, 48 and 72 h post feeding. However, in the case of insects infected with ESALQ 818, survival rates of blood fed insects returned to normal (sucrose fed levels) 96, 120 and 144 h after a blood meal (p > 0.01). When using pair-wise comparisons of sucrose fed and blood fed females at 96 h, the survival curves (Figure 2) were not significantly different (χ^2 ^2.59; df = 1; *P *= 0.1073).

When considering values for S_50 _, sucrose fed insects showed 50% survival/mortality in 3 days, whilst blood feeding resulted in an increased S_50 _value. At time zero S_50 _was 5 days for LPP 133 and 6 days for insects exposed to fungi at 24 h and 48 h post-blood feeding. For LPP 133, S_50 _values returned to sucrose fed levels from 72 h post-blood feeding onwards (S_50 _= 3 days), although analysis of variance showed that there was still a significant difference between blood-fed and sucrose-fed insects at this time. The daily survival rates of mosquitoes exposed to LPP 133 and ESALQ 818 following different feeding regimes are shown in Figures 1 and 2 respectively. When considering infection by LPP 133 of Rockefeller females maintained on sucrose or exposed to conidia 72-144 h after blood feeding, mortality was recorded from day 1 of the experiment. Whereas for females exposed to conidia 0 to 48 h after blood feeding, the first dead insects were seen only from day 3 of the experiment. A similar pattern of survival was seen for Rockefeller females exposed to ESALQ 818 (Figure 2).

**Figure 3:**
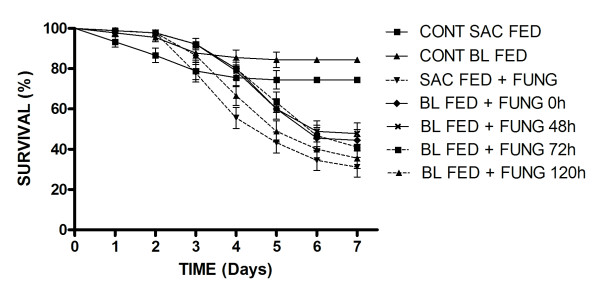
**Survival curves of blood or sucrose fed female *Aedes aegypti *(wild strain) exposed to *M. anisopliae *ESALQ 818 at different times post-feeding**. The control survival curve (Control BL FED) is the mean of the controls for each blood fed group (4 groups) for each time period post-feeding. Results are the means of three experiments for each treatment with 20 insects used per experiment. Data points without standard error bars had errors equal to that of the previous data point.

Experiments were repeated using wild strain F1 females, however, due to the limited number of eggs collected in the field, only one isolate, ESALQ 818, and three time periods post-blood feeding were tested. All fungus exposed insects, independent of their feeding regime, showed significant reductions in survival rates when compared to control groups (*p *<0.01). As seen previously for *A. aegypti *Rockefeller strain, blood feeding also significantly altered survival rates (mean percentage survival) of wild strain females when compared to their sucrose fed counterparts (F_(6,20) _= 71.90; *p *< 0.01; Table 2). When using a pair-wise comparison of sucrose fed and blood fed females at time zero, the survival curves (Figure 3) were significantly different (χ^2 ^6.283; df = 1; *P *< 0.0001). However, mosquitoes exposed to ESALQ 818 120 h after a blood meal were equally susceptible to the fungus as sucrose-fed insects (*p *> 0.01). When using pair-wise comparison of sucrose fed and blood fed females at 120 h, the survival curves (Figure 3) were not significantly different (χ^2 ^1.256; df = 1; *P *= 0.2624). As there were not sufficient insects to test a 96 h post-feeding, we cannot be sure that mosquitoes had not returned to normal levels of susceptibility before 120 h.

**Table 2:** Survival of female *A. aegypti *(wild strain) fed on sucrose or blood and subsequently exposed to ESALQ 818 at different times post feeding

Treatments	% Mean Survival ± SD	S_50 _
CONT SUC	74.4 ± 3.03 b	NA
CONT BL	84.4 ± 2.51 a	NA
SUC FED + ESALQ 818	34.4 ± 8.32 e	5
BL FED 0 h + ESALQ 818	44.4 ± 6.96 cd	6
BL FED 48 h + ESALQ 818	46.6 ± 6.39 c	6
BL FED 72 h + ESALQ 818	41 ± 6.00 d	6
BL FED 120 h + ESALQ 818	35.5 ± 7.16 e	5

Note: following the initial blood feed, mosquitoes were offered sucrose on a daily basis.

Results followed by the same letter indicate no significant differences when using Duncan's *post-hoc *test (5% probability). Values for S_50 _were calculated using Kaplan-Meier survival analysis. CONT: control; SUC: sucrose; BL: blood; NA: not applicable.

When using the criteria median survival (S_50_), the same pattern was seen as that for mean percentage survival with a rapid alternation in S_50 _to 6 days and a return to 5 days at 120 h post-blood feeding. The daily survival rates of wild strain mosquitoes exposed to fungi following different feeding regimes are shown in Figure 3. The survival curves of these females were different from those of Rockefeller strain females in that mortality of insects exposed to fungi, independent of the feeding regime, was only observed from day 3 of the experiment. Yet again it should be noted that there was a distinct difference in survival rates of blood-fed and sucrose-fed controls. Furthermore, it should be noted that wild strain females independent of the feeding regime, are less susceptible to fungal infection than Rockefeller strain females (compare Table 1 with Table 2).

## Discussion

Ideally, entomopathogenic fungi should be virulent to their insect hosts independent of host age, sex and physiological state. However, as seen here, nutritional state can significantly affect host susceptibility. Female *A. aegypti *that had ingested a blood meal rapidly became less susceptible to infection by *M. anisopliae *than sucrose fed females. This reduced susceptibility persisted for a duration of approximately 96 h, at which time survival rates returned to levels seen for sucrose fed females exposed to fungi.

These results are similar to those published by Mnyone et al. [17] when comparing blood-fed and glucose-fed *An. gambiae *, although no alterations in survival times were observed when exposing mosquitoes to fungi 3 h post-blood feeding. An alteration in survival rates (increased survival) was observed at 12 h and 36 h post-blood feeding. According to these authors, there were no differences in the survival of sugar-fed and blood-fed females 72 h post-feeding.

Mnyone et al. [17] suggested that reduced host susceptibility could be due to a putative increase in nutrient reserves of blood fed insects when compared to sugar-fed insects, thus delaying the detrimental effects of the fungus on the host, which in turn resulted in increased survival rates. However, up-regulation of the immune system following blood feeding could be responsible for the alteration in susceptibility.

In order to identify the possible causes for the reduced susceptibility of blood-fed female mosquitoes it is necessary to understand the fungal infection process. Entomopathogenic fungi normally infect their host by penetrating the integument, a well studied process that involves both physical and chemical (enzymatic) mechanisms [18].

The infection process of entomopathogenic fungi can be separated into three phases: (a) adhesion and germination of the conidia on the insect's cuticle; (b) penetration of integument by the germ tube, in order to invade the haemocoel and (c) development of the fungus within the haemolymph (colonization) resulting in host death [18]. There is a time lag between initial contact with the fungus and haemolymph colonization. Conidial germinating can be seen from 8 h under laboratory conditions (unpublished data: RIS) and as the earliest host-death can be seen after 24 h, cuticle penetration thus takes up to 16 h in highly virulent isolates.

Even before reaching the haemolymph, the insect will have already activated an immune response to the microbial invasion. Insect defenses are (a) physical barriers, such as the cuticle and peritrophic membrane, epithelial barriers, some of which carry specific immune mechanisms; (b) protease cascades leading to coagulation and melanization; (c) cellular responses such as phagocytosis and encapsulation, and (d) production of antimicrobial peptides and reactive oxygen species [19].

It is known that blood feeding in combination with sucrose significantly increases the melanization cascade in *An. stephens*, whereas insect fed on a sucrose only diet displayed a low immuno-competence [20]. In the current study, *A. aegypti *females were fed blood once, and subsequently offered sucrose *ad libitum *, which may have increased immune activity of the mosquitoes. It is common to provide sugar solutions for maintaining mosquitoes in laboratory studies; however, field captured *A. aegypti *females were shown not to have fed on sugars, indicating that this energy source is not necessary for survival and reproduction [21-23].

Studies of the effects of blood feeding on the longevity of mosquitoes have been contradictory. One hypothesis is that mosquitoes that feed on blood have lower survival rates due to a diversion of nutrients to egg production [16]. Other experiments reported that blood feeding increased the survival of mosquitoes, compared with those feeding only on sugar [15,24,25]. In the present study a slight increase in survival of blood-fed females was seen when compared to sucrose only fed females.

Blood fed *An. gambiae *suffered a series of physiological changes such as expression of genes related to formation of the peritrophic matrix, digestion, immunity and egg development [26]. Insect antimicrobial peptides and proteins take 1-3 hours to produce and 12 to 48 hours to reach their peak [27]. In the current study females became susceptible again to the fungus 96 hours after blood feeding, possibly when due to down-regulation of the immune system returning to the pre-blood meal levels.

The selection of highly virulent fungi is important for the control of *A. aegypti *especially as the dengue virus develops rapidly. On the seventh day, the females become infective [28] and transmit the virus following each blood meal [29]. The lethal effects of the entomopathogenic fungus should therefore occur before the parasite develops infective forms.

In the current study, on the seventh day of evaluation, there was significant mortality of *A. aegypti *fed on sucrose or blood, emphasizing the potential of the fungus for mosquito control. The highest value for S_50 _was 6 days, resulting in 50% vector mortality before the final development of the Dengue virus [29]. There were no differences in the values of S_50 _for LPP133 when compared to isolate ESALQ 818, except for zero and 72 hours post-blood feeding.

Blood fed wild strain females exposed to ESALQ 818, had significantly higher survival rates compared with females fed with sucrose except 120 h post-blood feeding. The value of S_50 _of wild strain females fed with sucrose was higher compared with mosquitoes reared in the laboratory (Rockefeller strain), 5 and 3 days respectively. It is probable that wild strain females are subject to selection pressure which results in greater genetic diversity and increased immunity, compared with females reared in the laboratory.

Reduction in mosquito susceptibility to fungi following a blood meal is of concern, thus females resting on fungus impregnated surfaces immediately after blood feeding will have a reduced risk infection. However, host seeking female *A. aegypti *remain highly susceptible to fungal infection. It should be remembered that reduced susceptibility is only a temporary phenomenon and may not alter the long term effects of fungi on the mosquito population. These results highlight the need for further studies on the insect-fungus interaction at a cellular and molecular level.

## Materials and methods

### Maintenance of Insect Colonies

*A. aegypti *(Rockefeller strain) colonies were reared in cages at 25°C; 75% RH;16:8 L/D photoperiod and provided with a 10% sucrose solution. Insects were provided with blood meals by placing a mouse, immobilized in a wire mesh bag, in the adult mosquito cages. Following the blood meal, oviposition occurred in beakers half filled with water and lined with filter paper placed in adult cages. Egg eclosion was stimulated by total immersion of the filter paper in water to which mouse food had been previously added (24 h) to reduce oxygen levels.

Larvae were maintained in plastic trays and fed on minced commercial mouse food until reaching the pupal stage. Pupae were separated into water filled beakers and transferred to cages before adult emergence. Recently hatched (2-3 days old) females were used for all experiments.

F1 wild strain adult female mosquitoes used in experiments here were obtained following collection of *A. aegypti *eggs laid in ovi-traps distributed around the campus of the State University of North Fluminense.

### Fungal Isolates and preparation of suspensions

The isolates of *M. anisopliae *used here were obtained from the collection at ESALQ (ESALQ818) in Piracicaba (São Paulo) and the Laboratory of Entomology and Plant Pathology at the State University of North Fluminense (LPP133) which had been previously demonstrated to have high virulence against adult *A. aegypti *[11]. Fungi were cultured on Sabouraud Dextrose Agar (Dextrose 10 g; Peptone 2.5 g; Yeast Extract 2.5 g; Agar 20 g in 1 L H_2 _0) at 27°C for 15 days before being used in experiments. Fungal suspensions were initially prepared in Tween 80 (0.05% in sterile distilled water) and conidial concentration determined using a Neubauer hemocytometer. A final concentration of 1 × 10^9 ^conidia mL^-1 ^was prepared by serial dilution. Fungal suspensions were vortex mixed vigorously before applying 1 mL to each side of a piece of filter paper (8 × 6 cm) using a Potter tower giving a conidial coverage of 1.5 × 10^8 ^conidia cm^2 ^on each side of the filter paper as previously described by Paula et al. [12].

Following spraying, the filter papers were left to dry for 16 h before being placed in plastic pots covered with netting (12 cm diameter × 7 cm high), into which mosquitoes were later placed. The filter paper was positioned upright in the pot. Thus, the total time the mosquitoes spent in actual contact with the filter paper can not be determined by this method as mosquitoes could choose not to land on the filter paper.

### Adult feeding and fungal infection regime

Two to three day old female mosquitoes were either offered 10% sucrose or blood fed on mice before being exposed to filter papers impregnated with *M. anisopliae *prepared as stated above. Only mosquitoes that had taken a blood meal (visual observation) were used in experiments. Mosquitoes were exposed to the fungi immediately after blood feeding (time zero) or exposed to fungi 24, 48, 72, 96, 120 and 144 hours post-feeding. Following exposure to fungi for 48 h, the mosquitoes were transferred to fresh pots. All groups were offered filter paper discs soaked in 10% sucrose and placed on the netting surface of the pots on a daily basis. Survival of insects was determined on a daily basis for a 7 day period and dead insects were removed during observations.

All experiments were carried out three times with a minimum of 30 insects per treatment or control group. The homogeneity of the replicate experiments was determined using the Log-rank Test [30] at the 95% significance level and subsequently the results were pooled to calculate mean mortality and standard deviation.

### Mean percentage survival and survival curve analysis

Mean percentage survival at seven days post-treatment was calculated and the results compared using one-way analysis of variance and Duncan's *post-hoc *test. Median survival time (S_50 _) was calculated using Kaplan-Meier survival analysis and pair-wise survival curve comparisons were carried out using the Log-rank (Mantel-Cox) test.

## Competing interests

The authors declare that they have no competing interests.

## Authors' contributions

ARP helped to carry out the experiments, participated in the design of the study and performed the statistical analysis. ATC helped carry out experiments and maintained the insect colonies. CPS participated in the design of experiments and writing of the manuscript. RIS conceived the study, participated in its design, supervised the experiments and wrote the manuscript. All authors read and approved the final manuscript.
